# Induction of Semi-Dwarf Trait to a Three Pistil Tall Mutant Through Breeding With Increased Grain Numbers in Wheat

**DOI:** 10.3389/fgene.2022.828866

**Published:** 2022-02-08

**Authors:** Ahsan Irshad, Huijun Guo, Shoaib Ur Rehman, Jiayu Gu, Rana Imtiaz Ahmed, Manzoor Hussain, Ali Ammar, Imtiaz Ali, Akash Zafar, Chaojie Wang, Chunyun Zhou, Lin Qiu, Luxiang Liu

**Affiliations:** ^1^ National Engineering Laboratory of Crop Molecular Breeding, National Center of Space Mutagenesis for Crop Improvement, Institute of Crop Sciences, Chinese Academy of Agricultural Sciences, Beijing, China; ^2^ Institute of Plant Breeding and Biotechnology, Muhammad Nawaz Sharif University of Agriculture, Multan, Pakistan; ^3^ Regional Agricultural Research Institute, Bahawalpur, Ayub Agricultural Research Institute, Faisalabad, Pakistan

**Keywords:** three pistil, heterosis, semi dwarf, synthetic hexaploid, breeding

## Abstract

Multi-ovary wheat (three pistil) is a unique germplasm for the seed production of hybrid wheat. The purpose of the present study was to transfer the multi-ovary trait to semi-dwarf plants to increase the production of grains in wheat crops. Therefore, tall, semi-dwarf, and dwarf plants were crossed with plants with the three-pistil trait. A three-pistil tall plant was used as the female parent, while tall (Synthetic hexaploid), semi-dwarf, and dwarf plants were used as male parents. F1 and F2 progenies with parents were planted in 2015-16 using RCBD. The outcome of the crosses showed that multi-ovary tall plants gave significant difference for all five traits (days to maturity, plant height, number of seeds per spike, grain weight per spike, and grain yield per unit area) in both generations. The greatest number of grains per spike and grain yield per unit area were obtained from the cross of three-pistil tall and dwarf parent (P1/P6) in the F1 and F2 generations. The cross also resulted in a significant reduction in height (96 cm). Further heterosis studies conducted with crosses between three-pistil tall and dwarf parent (P1/P6) showed the greatest heterosis and heterobeltiosis for the number of grains per spike (60.0 and 26.19%, respectively) and grain yield per m^2^ (27.68 and 2.85%, respectively). In the case of grain weight per spike, the heterosis value was also positive and significant (17.7). Meanwhile, for other traits, their values were negative for heterosis and heterobeltiosis. High numbers of grains and grain weight were found to be associated with positive heterobeltiosis and in turn the grain yield per m^2^, but plant height and maturity had negative affirmation with heterobeltiosis. Heterosis studies also indicated the dominant gene action for the three-pistil trait. Thus, the study clearly signified that grain yield can be increased by using the multi-ovary genotype with the semi-dwarf height. This new germplasm will be helpful for breeders to increase the production of wheat crops in the southern climate of Pakistan.

## Introduction

Wheat (*Tritium aestivum* L.) is consumed as a major staple food in almost all regions of the world and is grown on millions of hectares of land. Several products of wheat are also consumed by humans in different parts of the globe. One important product is Chapatti which is used as a staple food in Asian countries (Shitsukawa et al., 2009; Irshad et al., 2021). It accounts for approximately 30% of global grain production, while it provides 20% of the calories and essential amino acids to the human population (Howel et al., 2014). As the population of the world is increasing, the demand for wheat (as a food) is also on the rise. During the green revolution only one trait, i.e., plant height was improved. Similarly, for the next green revolution, it will be necessary to continue to increase production by improving the yield-related traits of wheat to meet the future demands of food security. To improve the yield potential of wheat, it is necessary to increase the grain number per spike and unit area (Frederick and Bauer, 1999). For this purpose, a wide range of genetic variations are required in the morphological structure of wheat to achieve high grain numbers per spike (Yang et al., 2015).

The female reproductive part is comprised of a multilocular ovary containing ovules, two filamentous styles, and a feathery stigma, while for the male reproductive part, a filament and an anther constitute a stamen where the pollen grains are present in the anther (Zhu et al., 2019). The three-pistil (TP) trait in wheat is a very important trait, in which 2–3 ovaries (well developed) in a floret are observed. The potential of yield in wheat can be improved by increasing the number of seeds in a spike. Therefore, wheat breeders have focused on morphological and genetic variability for yield-related traits (Irshad et al., 2021). The TP trait was first reported by Chen et al. (1983), which was an excellent line to increase wheat yield. The previous genetic studies revealed that the TP trait is controlled by a single dominant locus *pis1* (Peng et al., 2004) and located at chromosome 2D. Yang et al. (2011) identified seven differentially expressed genes in TP mutants using molecular technologies. The overexpression of the gene is attributed to the development of TP. Thus, it is imperative to use three-pistil germplasm in hybridization, which can give encouraging results.

Breeding programs as an essential approach to enhance crop yield and other quality parameters (Fu et al., 2014). A hybrid offspring which is the outcome of two genetically diverse individuals is superior to that of the mean of the parents (heterosis) or the better parent (heterobeltiosis). This phenomenon has been successfully utilized in fiber, cereals, and oilseed crops (Ahmad et al., 2014). . Intriguingly, in a self-pollinated crop like wheat, heterobeltiosis is much more desired than heterosis in any breeding program (Zaazaa et al., 2012). Although the hybridization of wheat has achieved significant progress (Boeven et al., 2016), there is a lack of large-scale effective utilization of wheat hybrids. The low propagation coefficient of hybrids in wheat leads to a higher cost of seed production. This proves to be a bottleneck for the efficient utilization of hybrid wheat.

In the present study, a TP tall mutant was crossed with normal tall, semi-dwarf, and dwarf genotypes. The foremost objective of hybridization was to transfer the TP trait into wheat germplasm cultivated in Pakistan and increase the grain number per spike for semi-dwarf and dwarf height plants, to help increase wheat production.

**FIGURE 1:**
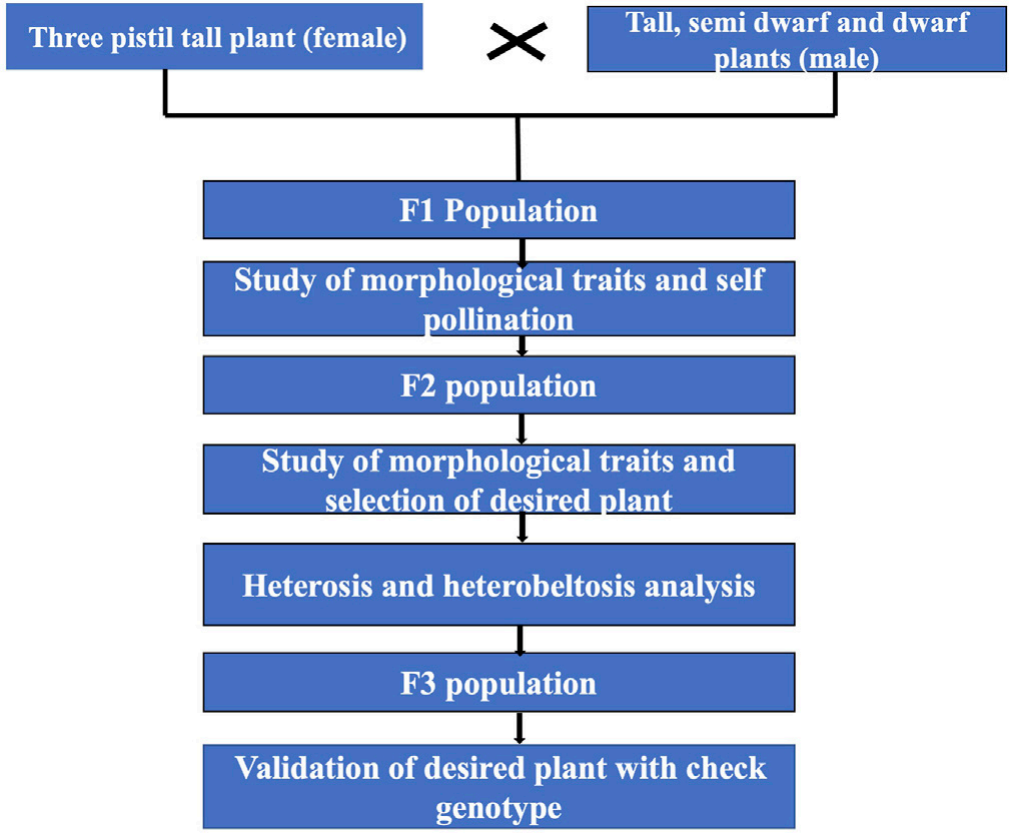
Schematic diagram for selection of desired semi-dwarf three-pistil plant.

The other objective was to develop baseline parental material to be used as a genetic resource and in hybridization programs to create genetic diversity. The multi-ovary trait can be exploited for hybrid wheat development as the parents exhibit significant heterotic effects or may be used to develop conventional wheat varieties by repeated cycles of selection in advance filial generations, which is also evident from the results. The overall scheme of the work is given in Figure 1.

## Material and Methods

### Plant Material and Construction of Research Population

This experiment was conducted at the Regional Agricultural Research Institute Bahawalpur, Pakistan. The goal was to develop new plants carrying the multi-ovary trait with desirable maturity (medium duration), number of seeds per spike, and height. The multi-ovary genotype (tall) was collected from the Wheat Wide Crosses Laboratory, National Agriculture Research Centre, Islamabad, and it was crossed with tall (normal ovary), semi-dwarf (normal), and dwarf (normal).

Tall 3-pistil parent was used as the female parent in all the crossing schemes, which are as follows:

1) Three-pistil × tall 1 (normal)
2) Three-pistil × tall 2 (normal)
3) Three-pistil × semi-dwarf 1 (normal)
4) Three-pistil × semi-dwarf 2 (normal)
5) Three-pistil × dwarf 1 (normal)
6) Three-pistil × dwarf 2 (normal)

**Table:** 

Parents	Name of parents
P1 (Three-pistil and tall)	Yr-Pastor-10
P2 (Normal and tall 1)	SH Bahawalpur
P3 (Normal and tall 2)	SH-220
P4 (Normal and semi-dwarf 1)	Mairaj-08 (A commercial wheat variety)
P5 (Normal and semi-dwarf 2)	V-6309 (Advance strain)
P6 (Normal and dwarf 1)	TDB**-1
P7 (Normal and dwarf 2)	TDB-2

* SH , synthetic hexaploid, **TDB , triple dwarf from bahawalpur.

The parents were sown in 2014–2015 and the crossing was done in February 2015 to get the F1 seeds. F1 and F2 were planted during 2015–2016. The F2 seeds were produced by the planting of F1 at Kaghan (off-season site) during 2016, and F3 was planted during 2016–2017. The sowing of F1 and F2 was done in triplicate RCBD with 5 m of single row length. The plant to plant distance was 15 cm. The parents were also sown along with F1 and F2.

### Morphological Analysis

A total of 30 plants from the F1 population from each cross and 75 plants from the F2 population of each cross were selected randomly for data recording and analysis. Days to maturity, plant height, number of grains per spike, grain weight per spike, and grain yield per m^2^ were recorded. The mean values were used to characterize the corresponding traits. Photographs of the parents and selected plants were taken using Nikon D600 digital camera (Nikon, Tokyo, Japan), whereas those of the pistils were taken using a Nikon E995 digital camera (Nikon, Tokyo, Japan).

### Analysis of the Genetic Basis of the Multi-Ovary Trait

The multi-ovary trait was measured during the flowering season and maturation stage, and the trait of each individual of the F1 and F2 populations was measured. The numbers of ovaries and seeds in the florets of each spike were counted. Each plant with a floret carrying more than one ovary during the flowering period and setting more than one seed at the maturation stage was recorded as a multi-ovary plant, while plants with florets producing one ovary and setting one seed were recorded as mono-ovary plants, a classification that followed that of Peng et al. (2004) and Ma et al. (2006).

For the F1 generation, all 30 plants were measured. For each of the F2 populations, 75 plants were randomly selected for measuring. The numbers of multi-ovary and mono-ovary plants were counted. For each population of the F3 generations, all plants were measured to determine whether the multi-ovary trait segregated in each population. The number of different segregations of the multi-ovary trait in each population was counted.

### Statistical Analysis

The recorded data were analyzed statistically by using the technique as given by Steel et al. (1997). In F3, the selected genotypes were compared with local checks, i.e.; Fareed-06 for all four traits.

Also, heterosis, heterobeltiosis, and inbreeding depression were computed following Matzinger et al. (1962) and Fonsecca and Patterson, (1968). The following formulae were used for heterosis and heterobeltiosis in each environment for all the characters studied:

Heterosis over mid parent (H %) = ((F1-MP)/MPX100)
  SE (F1-MP) = (3Me/2r) 1/2
Heterosis over better parent (HB %) = ((F1-BP)/BPX100)
  SE (F1-BP) = 2Me/r)1/2
Inbreeding depression (ID %) = ((F1-F2)/F1X100)
  SE (F1-F2) = 2Me/r) ½

Where Me = mean squared error; MP = mean mid parent value; BP = mean better parent value; R = number of replications. Standard error values were used to elucidate the significance of heterosis and inbreeding depression for each character expression under different environments.

## Results

### Morphological Analysis

**FIGURE 2:**
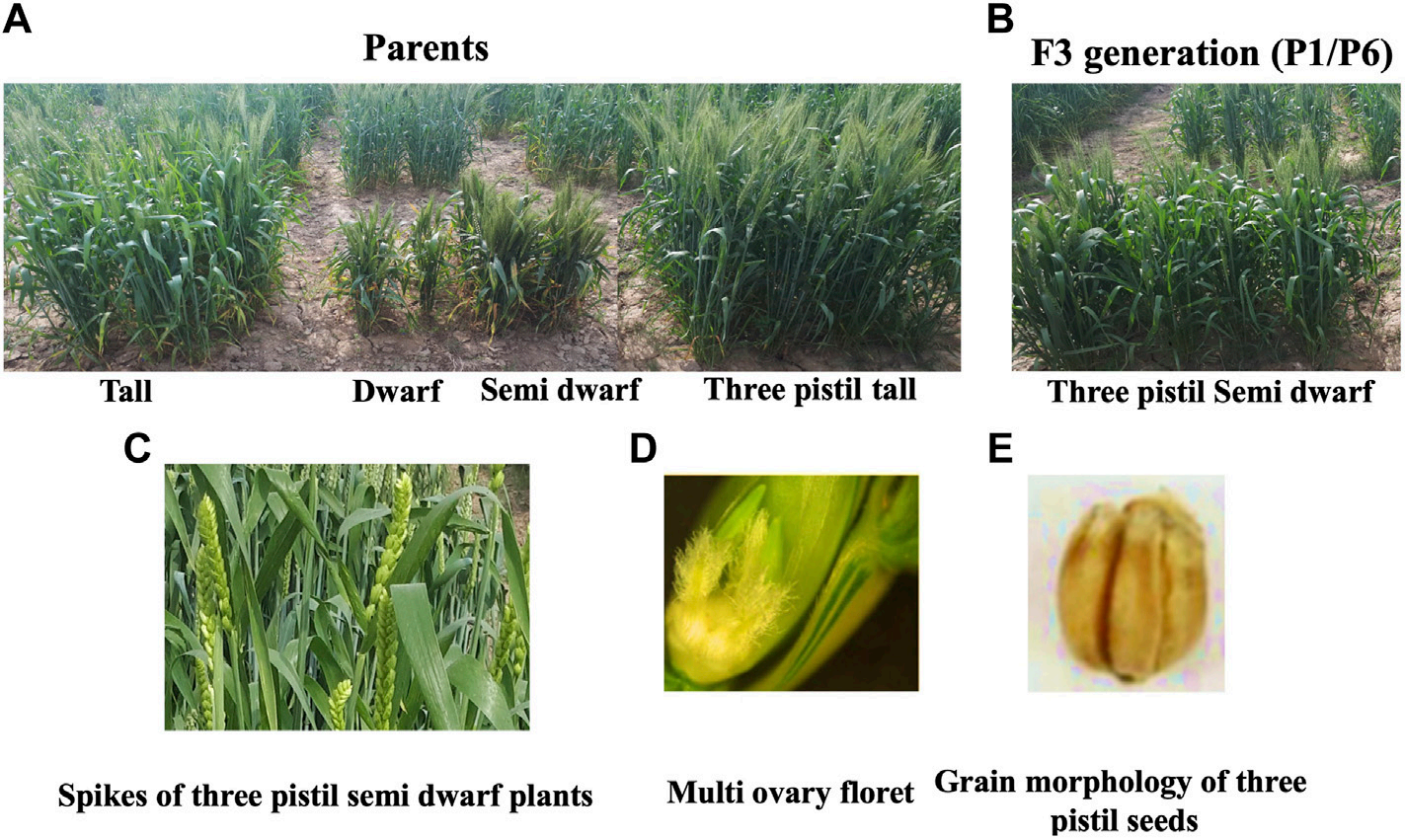
Phenotypic characterization of mono ovary and multi ovary plants. **(A)** Parents used in crosses. **(B)** Selected semi-dwarf three-pistil plant in F3 generation. **(C)** Spikes of three-pistil plants. **(D)** Three-pistil in semi-dwarf plant. **(E)** Mature grains of the three-pistil semi-dwarf plant.

**FIGURE 3:**
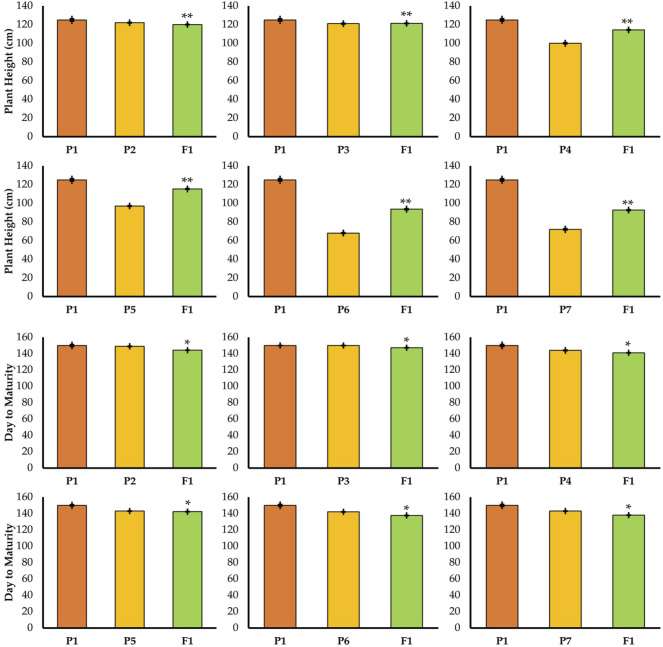
Mean performance of parents and F1 hybrid for plant height and day to maturity traits. * = indicates significant from one parent at 0.01 probability level; ** = indicates significant from both parent at 0.01 probability level.

**FIGURE 4:**
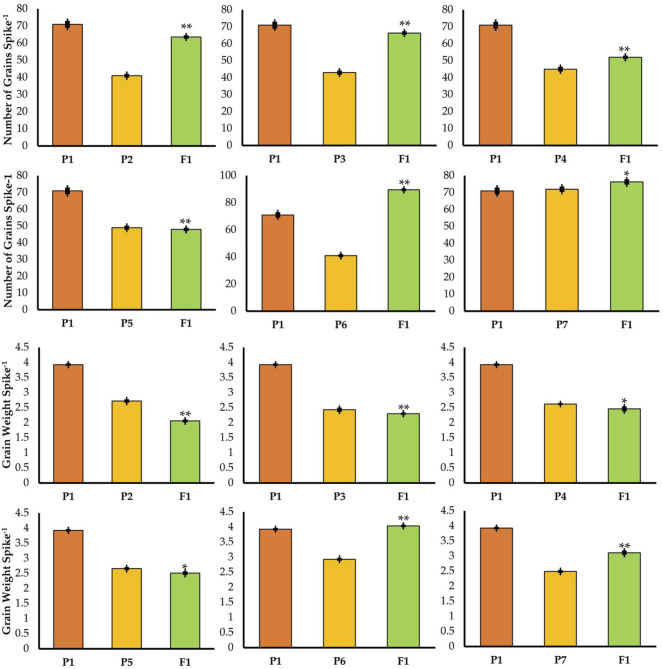
Mean values of F1 with their parents in different crosses for number of grains per spike and grain weight per spike. * = indicates 5% probability level; ** = indicates 1% probability level.

Different parents were used which were significantly different in plant height in the crosses (Figure 2). On the basis of these crosses, the best cross was selected with semi-dwarf height and the TP trait (Figure 2). Statistical analysis of the data indicated significant differences for all traits in the F1 and F2 generations (Figures 3, 4; Table 1). The trait means of the parents, and of F1 with their parents are given in Figures 3, 4, while the mean square of F2 is given in Table 1.

**TABLE 1:** Mean squares of different traits in F2. * = indicates 5% probability level; ** = indicates 1% probability level.

Sr.No	Traits	F2 generation		
		Rep	Genotype	Error
1	Days to maturity	1.5	524.3*	5.5
2	Plant height (cm)	.6	3.6**	3.0
3	No. of grains per spike	1.7	5.2**	2.5
4	Grain weight per spike (g)	.3	7.0**	1.0
5	Grain yield (kg/m^2^)	.9	2.3**	2.1

Firstly, all the yield-related traits had been studied in the parent lines. The results are given in Figures 3, 4. Theheight of the TP plants was 150 cm while the semi-dwarf and dwarf were 97 and 72, respectively. Similarly, there was a significant difference in grain number, in which the TP plants had 71 grains per spike, but the dwarf and semi-dwarf plants had 41–45 grains per spike.

### Days to Maturity

The cross between TP tall and normal tall plants matured in 144.3 days in F1 and 143 days in F2 (Figure 3) which indicated little reduction of duration. A similar trend was also observed in cross P1/P3. When TP tall was crossed with semi-dwarf 1, the F1 and F2 generations matured in 142.3 and 140 days, respectively. There was no significant change in maturity. Likewise, the results of the P1/P5 cross (TP and dwarf 1) for F1 and F2 showed 137.6 and 136 days to mature, respectively. The same situation was noticed in F1 and F2 for the cross between TP and dwarf 2. It showed that there was a reduction in maturity duration in both the F1 and F2 generations.

### Plant Height

The results for the P1/P6 cross (TP and dwarf 1) in F1 and F2 resulted in plants having 93.3 and 90 cm height, respectively. Similar findings were noticed in F1 and F2 for the cross between TP and dwarf 2. These results show that there was a reduction in plant height in both generations; F1 and F2 (Figure 3). The plants selected in F1 and F2 from the cross of three-pistil and dwarf gave plants with desirable height (semi-dwarf).

### Number of Grains per Spike

In F1 and F2 generations of the cross between TP tall and normal tall 1, the grain numbers recorded per spike were 63.6 and 60, respectively, while in the case of the cross between three-pistil and normal tall 2, the grain numbers were 66.3 and 62 in F1 and F2, respectively (Figure 4). For both of these crosses, grains per spike was increased but the plants did not show a desirable height, as shown in Figure 3. Thus, the crosses further continued and a TP tall was pollinated with semi-dwarf male parents (Mairaj-o8 and V-6309), where F1 and F2 yielded 52–48 and 49–46 grains per spike, respectively. Similarly, more grains were obtained from a cross between the three-pistil tall line with a dwarf parent where the grain numbers increased to 89.6 in F1 and 78.2 in F2 (Figure 4).

### Grain Weight

The grain weights per spike were 2.06 and 2.00 in F1 and F2, respectively, in the cross between TP tall and normal tall 1. The cross between TP tall and normal tall 2 had grain weights of 2.30 and 2.10 g in F1 and F2, respectively (Figure 4). The crosses between TP tall and semi-dwarfs 1 and 2 (Mairaj-08 and V-6309) yielded grain weights per spike of 2.46 and 2.51 g in F1 and F2, respectively, for the former, and 2.33 and 2.41 g in F1 and F2, respectively, for the latter. The most desirable cross appeared from a TP tall plant pollinated with dwarf parents. This cross resulted in 4.04 and 4.00 g in F1and F2, respectively, which is the greatest grain weight obtained (Figure 4).

### Grain Yield per m^2^

**FIGURE 5:**
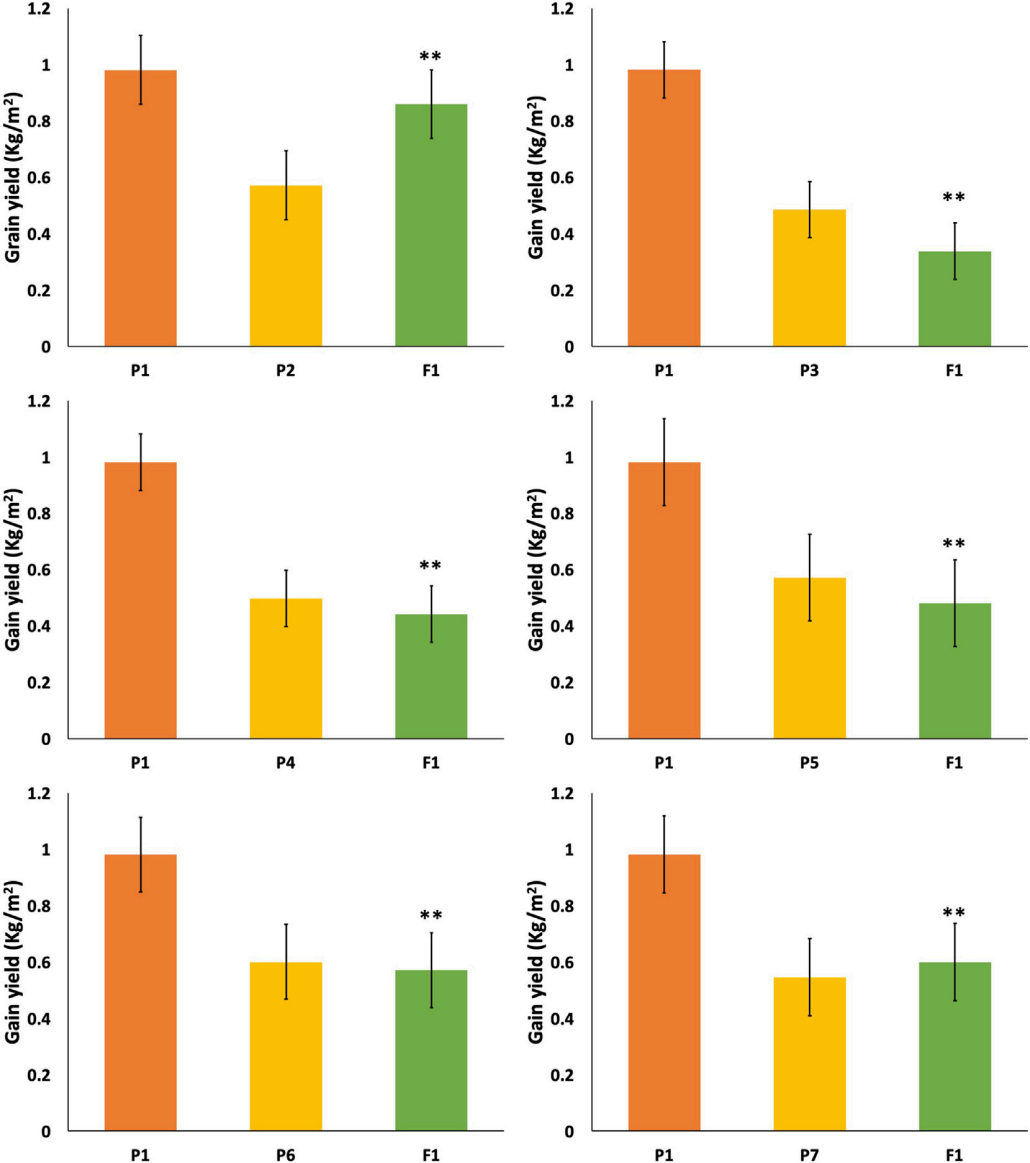
Mean performance of parents and F1 hybrid for grain yield (kg/m^2^). * = indicates significant from one parent at 0.01 probability level; ** = indicates significant from both parent at 0.01 probability level.

The cross between TP tall and dwarf gave a yield of 1.01 kg/m^2^ in F1 and 0.8 kg/m^2^ in F2 (Figure 5). There was a significant change in grain yield per unit area. Whereas the results of a cross P1/P2 (TP and tall) showed 0.432 and 0.38 kg/m^2^ in F1 and F2, respectively.

### Heterosis and Heterobeltiosis

Five traits (days to maturity, plant height, number of grains per spike, grain weight per spike, and grain yield per m^2^) were studied for heterosis and heterobeltiosis. For days to maturity, crosss P1/P3, P1/P4, and P1/P6 gave highly significant superiority of F1, while cross P1/P2 showed negative heterosis in F1. The feasibility of exploitation of heterosis is useful to determine the superiority of hybrids that are particularly better than parents. Heterobeltiosis was negative and significant for days to maturity in all crosses, which indicated that maturity remains stable. In F2, the greatest value of inbreeding depression was 1.61 for the cross P1/P5, and the least value was .88 for the cross P1/P2. With regard to plant height, the greatest heterosis was 3.7 for P1/P5 and the least was −6.2 for P1/P7, which indicates a significant change in plant height in the case of cross P1/P7. In heterobeltiosis all the values were negative, indicating a reduction in height in F1 (Table 2). Regarding inbreeding depression for plant height, all the crosses showed positive effects. No change in plant height was observed in F2. For grain weight per spike, the greatest heterosis was observed in cross P1/P6 (17.7) and the least was −39.7 in cross P1/P2 (Table 2). For the number of grains per spike, all the crosses were positive and significant. While considering inbreeding depression, there was no superiority in F2. Heterosis and heterobeltiosis for grain yield per m^2^ were significant but negative for all crosses except for the cross P1/P6 (27.68 and 2.85% in F1 and F2, respectively). So we can say the cross P1/P6 was the best cross with semi-dwarf height, high grains per spike, and high grain yield per m^2^.

**TABLE 2:** Heterosis (H, %), Heterobeltiosis (HB, %) and Inbreeding depression (ID%) for five traits.

Sr.No	Cross	Days to maturity			Plant height			No. of grain/spike			Grain weight/spike			Grain yield (kg/m^2^)		
		H	HB	ID	H	HB	ID	H	HB	ID	H	HB	ID	H	HB	ID
1	P1/P2	−3.6	−3.8	.90	−2.5	−4.0	1.67	13.50**	−10.40**	5.66**	−39.7**	−49.1**	2.91^NS^	−44.40**	−56.08**	12.03**
2	P1/P3	−1.8	−1.9	.88	−1.3	−2.9	1.07	16.01**	−6.60**	6.48**	−27.6**	−41.4**	8.69**	−38.96**	−54.37**	13.28**
3	P1/P4	−4.2	−6.0	1.41	1.6	−8.5	1.13	−10.8**	−26.70**	5.76**	−24.7^NS^	−37.4**	5.28**	−28.64**	−46.23**	16.15**
4	P1/P5	−3.0	−5.1	1.61	3.7	−7.7	2.86	−20.1**	−32.30**	4.16**	−23.7**	−36.1**	83.67**	−35.39**	−48.87**	3.98 ^NS^
5	P1/P6	−5.8	−8.2	1.16	−3.3	−25.3	3.53	60.0**	26.19**	12.94**	17.7**	2.8^NS^	.24^NS^	27.68**	2.85 ^NS^	20.79**
6	P1/P7	−5.8	−8.0	1.15	−6.2	−25.9	1.72	31.55**	7.46**	12.18**	−3.1**	−20.8**	3.21**	−16.88**	−35.33**	7.566**

** *, Significance at *p*.05 and *p*.01 levels, respectively. H, heterosis; HB, heterobeltiosis; ID, inbreeding depression.

### Confirmation of Yield Traits in the F3 Generation

**FIGURE 6:**
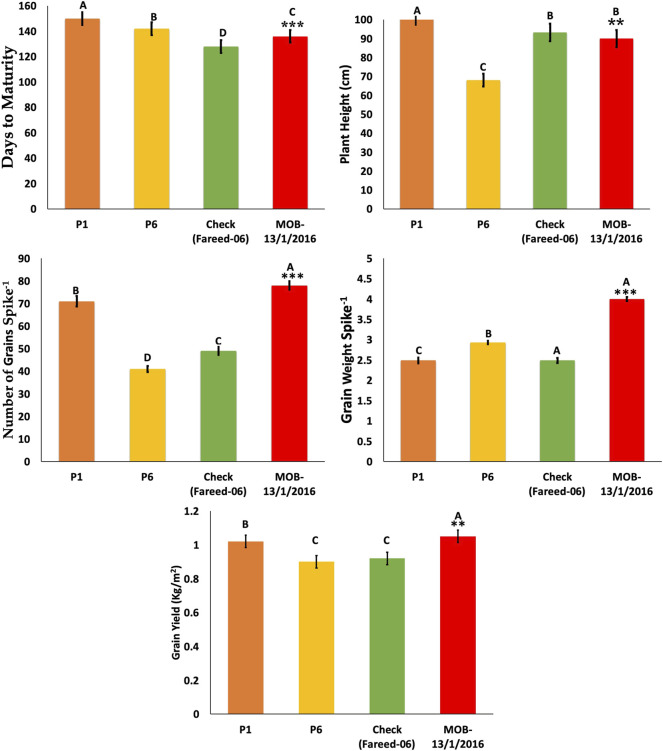
Comparison of selected crosses in F3 generation with commercial variety and parents. * = indicates significant from one parent at 0.01 probability level; ** = indicates significant from both parent at 0.01 probability level. *** = indicates significant from check at 0.01% probability level.

The selected cross from P1/P6 was given the name/MOB-13/1/2016 and was compared with check variety Fareed-06 in the F3 generation to validate the results. The line MOB-13/1/2016 was TP with desirable plant height (90 cm), a greater number of grains per spike, and significantly increased grain weight per spike and grain yield per m^2^ (Figure 6).

### Genetic Analysis of the Multi-Ovary Trait

The multi-ovary trait was segregated in the F2 and F3 population derived from the reciprocal crosses between multi-ovary tall and mono-ovary semi-dwarf and dwarf plants. As shown in Table 3, the F2 population was segregated into multi-ovary and mono-ovary plants. The Chi-squared test indicated that the segregation ratio of multi-ovary to mono-ovary plants was 3:1, which is the typical segregation ratio according to Mendel’s law in all crosses. This indicated that the multi-ovary trait is controlled by a single dominant gene.

**TABLE 3:** Genetic analysis of multi-ovary trait in F2 generation.

Combinations	Multi-ovary: Mono ovary	Theoretical ratio	X^2^	*p* Value (df = 1)
P1/P2	60: 15	3:1	.401	.521
P1/P3	58: 17	3:1	.399	.572
P1/P4	55:20	3:1	.3.87	.580
P1/P5	63:17	3:1	.415	.520
P1/P6	59:16	3:1	.400	.516

## Discussion

Maturity is an important trait for successful cropping. Some genotypes are of long duration, while some have a short duration. In spring wheat, up to 120 days to maturity is considered early maturity while more than 135 days to maturity is considered long duration in Pakistan (Masood et al., 2005). However, 135–150 days maturity is considered a desirable period. Long-duration genotypes are often exposed to high temperatures in the reproductive stage and lose grain weight which results in low production (Ain et al., 2015). The overall situation indicated that maturity showed a significant difference in the cross between TP and dwarf (P1/P6) but for all other crosses maturity was not influenced.

Plant height plays an important role in production, particularly when fertilizers are applied. Long maturity along with greater height causes low production due to lodging (Gulnaz et al., 2011). Generally, landraces are tall (115–120 cm), lower yield, and less disease tolerant and so cannot be applied for wheat breeding (Hasnain et al., 2006). Therefore, several outstanding modern cultivars with high yield and disease tolerant characteristics from Europe and America were widely adopted in breeding programs, such as Nanda 2,419 (i.e., Mentana, Italy), Ardito (Italy), Lovrin 10 and Lovrin 13 (Romania), Songhuajiang 2 (i.e., Minn 2,761, United States), Funo (Italy), Abbondanza (Italy), Orofen (Chile), and Gansu 96 (i.e., CI12203, United States) (Ain et al., 2015). Wheat breeding program in Pakistan started in the early 1930s and was accelerated after the Green Revolution. Earlier, the breeding program was focused on the selection of landraces with higher yield and disease tolerance (Khan et al., 2000). Later, it was extended to some other characteristics of wheat grain. The semi-dwarf group is characterized by a height range of 80–110 cm, while the dwarf group has a height range of 40–70 cm. In dry and low humidity areas, a height range of 85–100 cm is desirable. For selection, height is given more importance by breeders. Usually, shorter height is associated with better tillering, and taller height with low tillering. Thus, a three-pistil plant with reduced height is desired (Liu et al., 2020).

Grains per spike that is caryopsis contributes towards the high yield in wheat thus as such more grain number signifies more production (Mohsin et al., 2009). The number of grain is dependent on the number of spikelets present in a spike, and tillering capacity determines the later trait. It is an established fact that increasing the number of grains per spike reduces the number of spikes per plant due to negative correlation. The reduced spikes per plant can be compensated agronomically by increasing the number of spikes per unit area through adjusting seed rate. This aspect is also under consideration, and efforts are being made to incorporate high tillering capacity through hybridization. Wheat breeders tend to select plants with high numbers of grains per plant (Saleem et al., 2015). In multi-ovary plants, one floret carries three pistils that result in three grains, as compared to a normal floret which carries only one pistil. While utilizing the multi-ovary trait, the number of grains can be increased (Ayoub et al., 2019). So the plants/families in the cross between three-pistil tall and normal dwarf 1 (P1/P6) that had the three-pistil trait and medium height were selected and promoted to F3. Peng et al. (2004) used three-pistil plants to increase grain number. Dobrovolskaya et al. (2009) also suggested the use of TP plants for increasing grain numbers.

Besides grain number, another yield attribute/trait that holds significant importance for wheat production is grain weight. Though the multi-ovary trait gives rise to greater grain numbers, grain weight is affected by a large number of factors such as genotypes, soil moisture, planting methods. and floret size (Allah et al., 2010). Floret size in multi-ovary plants is also helpful for healthy grains. The cross of three-pistil tall pollinated with dwarf parents produced the following desirable traits in the progeny: fewer days to maturity, appropriate plant height, greater number of grains per spike, and greater grain weight (Anjum et al., 2017).

The genetic basis for the expression of heterosis superior to the parents has been described by Rasheed et al. (2016). In this study the number of grains per spike was the focus due to the three-pistil germplasm. The data regarding the number of grains per spike showed superiority over the best parent. The situation showed the dominance type of gene action for the three-pistil trait. Peng et al. (2004) and Tiwari et al. (2011) also reported dominance gene action for TP trait. The cross P1/P6 showed a high number of grains per spike that was significantly different from the parents (Table 3), which indicates that the TP trait was due to positive and non-significant heterobeltiosis of grain weight per spike and negative heterobeltiosis for plant height.

Based on this study it can be concluded that the TP germplasm should be used as the female parent while transferring the trait. Moreover, the development of TP semi-dwarf germplasm will be helpful for breeders to increase production. This study clearly suggests that heterosis studies showed the dominance type of gene action for the TP trait, and thus TP germplasm with medium height should be used as the female parent with a dwarf plant as the male parent in future breeding programs.

## Data Availability

The original contributions presented in the study are included in the article/Supplementary Material, further inquiries can be directed to the corresponding author.

## Fundings

The work is supported by the Crop Varietal Improvement and Insect Pests Control by Nuclear Radiation, China Agriculture Research System of MOF and MARA (CARS-03), and the Agricultural Science and Technology Innovation Program (CAAS-ZDRW202002) of China.
